# Identification of an aquaculture poriferan “Pest with Potential” and its phylogenetic implications

**DOI:** 10.7717/peerj.5586

**Published:** 2018-09-24

**Authors:** Adrian Galitz, Steve de C. Cook, Merrick Ekins, John N. A. Hooper, Peter T. Naumann, Nicole J. de Voogd, Muhammad Abdul Wahab, Gert Wörheide, Dirk Erpenbeck

**Affiliations:** 1 Department of Earth- and Environmental Sciences, Ludwig-Maximilians-Universität München, Munich, Germany; 2 Department of Zoology, School of Biological Sciences, Formerly: University of Auckland, Auckland, New Zealand; 3 Biodiversity Program, Queensland Museum, South Brisbane, Queensland, Australia; 4 Griffith Institute for Drug Discovery, Griffith University, Nathan, Queensland, Australia; 5 Naturalis Biodiversity Center, Leiden, The Netherlands; 6 Institute of Environmental Sciences, Leiden University, Leiden, Netherlands; 7 Australian Institute of Marine Science, Indian Ocean Marine Research Centre, The University of Western Australia, Crawley, Western Australia, Australia; 8 GeoBio-Center, Ludwig-Maximilians-Universität München, Munich, Germany; 9 SNSB—Bavarian State Collections of Palaeontology and Geology, Munich, Germany

**Keywords:** Aquaria sponge, *Collospongia auris*, *Lendenfeldia chondrodes*, Molecular taxonomy, Sponges, Demospongiae, Porifera, Keratosa, Aquaria, Model organism

## Abstract

Correct identification and classification of sponges is challenging due to ambiguous or misleading morphological features. A particular case is a blue keratose sponge occasionally referred to as the “Blue Photo Sponge” among aquarists, which appears frequently (and in several cases unintended) in private aquaria. This spicule-less species, occasionally specified as *Collospongia auris* Bergquist, Cambie & Kernan 1990, not only displays a high phenotypic plasticity in growth form and colour, it also proliferates in aquacultures under standard conditions unlike most other sponges. Therefore, this species is regarded as a pest for most aquarists. In turn, the ease of cultivation and propagation in aquacultures qualifies this species as a model organism for a wide array of scientific applications. For these purposes, correct identification and classification are indispensable. We reconstructed ribosomal gene trees and determined this species as *Lendenfeldia chondrodes* (De Laubenfels, 1954) (Phyllospongiinae), distant to *Collospongia auris*, and corroborated by skeletal features. Additionally, the resulting phylogeny corroborated major shortcomings of the current Phyllospongiinae classification—its consequences are discussed.

## Introduction

Animal identification at species level still relies predominantly on the use of diagnostic morphological characters, thus applying mostly morphology-based species concepts (Mayr, 1963; see e.g., De Queiroz, 2007). Strict morphological classifications can, however, lead to misidentifications and conflicting phylogenetic hypotheses, if clear-cut morphological characters are missing, or characters are misleading (Jenner, 2004). Sponges (Phylum Porifera) are particularly challenging as their morphological characters are often difficult to apply for species delineation. Traditionally, sponge taxonomy is based on skeletal features, which in most lineages consist of siliceous or calcareous spicules, and/or an organic fibrous skeleton. However morphological characters in sponges are subject to considerable levels of homoplasy, and unintentional pooling of two or more distinct species under a single name (cryptic species) can frequently occur (e.g., Reveillaud et al., 2010). Likewise, environmentally induced phenotypic plasticity, which may transform external (e.g. Swierts et al., 2013) *and* skeletal (e.g. Cárdenas & Rapp, 2013) morphologies to different morphotypes, can severely hamper unambiguous morphological identification.

Keratose sponges (Class Demospongiae: Subclass Keratosa) constitute a sponge group of phylogenetic (e.g. Erpenbeck et al., 2012b), biochemical (see e.g. Munro et al., 1999) and economical (bath sponges, e.g., *Spongia officinalis*) relevance. However, the absence of a spicule skeleton in Keratosa, fully replaced by organic (spongin) fibres, dramatically limits the suite of applicable morphological apomorphies and challenges species identification (see also Wörheide, 2008). This hinders the utilization of keratose sponges for scientific applications, including pharmaceutical bioprospecting.

A conspicuous keratose sponge species is occasionally referred to as “Blue Photo Sponge” (among other trivial names) in aquaristic online fora due to its distinctive bright blue/purple colour caused by cyanobacterial symbionts (see Osinga, Tramper & Wijffels, 1998). This sponge is dreaded by many aquaria enthusiasts as a pest and as a threat to other aquaria organisms due to its fast growth and resistance to most methods of removal once established in an aquarium system (Brümmer & Nickel, 2003; Knop, 2016). In turn, the apparent ease for cultivation and propagation in aquacultures (Osinga, Tramper & Wijffels, 1998; Brümmer & Nickel, 2003) also offers a wide array of possible developmental, functional, morphological, physiological, and environmental experiments. Despite its infamy among aquarists and its scientific potential, the taxonomy of this sponge is still uncertain, and occasional referrals as *Collospongia auris* (Bergquist, Cambie & Kernan, 1990; Fosså& Nilsen, 1996; Osinga, Tramper & Wijffels, 1998; Knop, 2016) await verification. This study aims to identify and classify this poriferan “Pest with Potential” and assess its phylogenetic position and genetic variation in order to provide a solid taxonomic foundation for all aspects of subsequent research on this easily cultivable species.

## Material and Methods

Fresh tissue samples were taken from our own research aquaria systems (Molecular Geo- and Palaeobiology Laboratory, Ludwig-Maximilians-Universität München) as well as from different sources, including various aquaria shops and -services (see “LAB” and “SHOP” in Supplemental Information). Comparative material of keratose sponges were based on various museum collections, particularly the Queensland Museum (QM). Since type material is the only truly reliable reference point for taxonomy, as it represents the original specimen after which a species is described, we included tissue samples of different holo- and neotypes from the Australian Museum (AM, Sydney, Australia) and from The Natural History Museum (BMNH, London, United Kingdom). For a complete list of samples see Supplementary Material.

### Morphological analyses

For histological analyses of the “Blue Aquaria Sponge” we crafted, after imbedding in paraffin, microtome sections between 15 and 400 µm thickness from small portions of fresh sponge tissue (specimens GW8181, GW30002 and GW30003), which subsequently were stained with Van Giesson and/or Masson Goldman dye to obtain a greater contrast of the spongin fibers. Microscopic analyses were performed on a Leica DMLB transmitted light microscope mounted with a Leica DFC 480 camera for digital imaging, which was used in combination with the Leica Application Suite (LAS, version 4.5) software.

### DNA extraction, amplification and sequencing

DNA extractions of fresh aquaria sponge material were obtained using Qiagen Spin Columns (DNeasy Tissue Kit; Qiagen, Hilden, Germany). For type material (*Collospongia auris, Carteriospongia foliascens*, *Strepsichordaia lendenfeldi*, an established protocol for CTAB (Cetyltrimethylammoniumbromide) extraction was used, which has been shown to be reliable for holotypes (Sambrook, Fritsch & Maniatis, 1989; see Erpenbeck et al., 2016a). For molecular identification of the samples by means of phylogenetic reconstructions, we used DNA markers successfully applied in earlier studies. The C-Region of the large nuclear ribosomal subunit (28S) is becoming a predominant marker for molecular taxonomy in sponges (Voigt & Wörheide, 2016 ; Erpenbeck et al., 2016b), and is recruited in this approach for initial classification. Furthermore, the highly variable nuclear ribosomal internal transcribed spacers (ITS) were sequenced due to their suitability in species-level phylogenetic reconstructions in keratose sponges (see also Wörheide, Nichols & Goldberg, 2004; Erpenbeck et al., 2012a; Abdul Wahab et al., 2014). Due to high amounts of bacterial and fungal associates to sponges, specific keratose sponge primers were designed in the process, for both the entire ITS region, and ITS-2 sub-region separately, as well as for the 28S C-region (see Table 1).

**Table 1:** List of primers used in this study, including the references for the specifically designed primers.

**Name**	**Nucleotide sequence**	**Target region**	**Origin**
RA2_keratose (fwd)	5′GRA TGG TTT AGT GAG ATC TT 3′	ITS	This paper, modified after Wörheide (1998)
ITS2.2_keratose (rev)	5′AAA TTC AGC GGG TAG YCT GG 3′	ITS	This paper, modified after Wörheide (1998)
5.8S_keratose (fwd)	5′TGA CAA CTT CTG ACG GT 3′	ITS	This paper, modified after Chombard, Boury-Esnault & Tillier (1998)
28S-C2_keratose (fwd)	5′GAA AAG AAC TTT GRA RAG AGA GTC 3′	28S	This paper, modified after Chombard, Boury-Esnault & Tillier (1998)
28S-D2_keratose (rev)	5′CCG TGT TTC AAG ACG GGT CGR ACG AG 3′	28S	This paper, modified after Chombard, Boury-Esnault & Tillier (1998)
RA2-fwd	5′GTC CCT GCC CTT TGT ACA CA 3′	ITS	Wörheide (1998)
ITS2.2-rev	5′CCT GGT TAG TTT CTT TTC CTC CGC 3′	ITS	Wörheide (1998)
5.8S-1-fwd	5′GTC GAT GAA GAA CGC AGC 3′	ITS	Chombard, Boury-Esnault & Tillier (1998)
28S-C2-fwd	5′GAA AAG AAC TTT GRA RAG AGA GT 3′	28S	Chombard, Boury-Esnault & Tillier (1998)
28S-D2-rev	5′TCC GTG TTT CAA GAC GGG 3′	28S	Chombard, Boury-Esnault & Tillier (1998)

Amplifications were conducted in 12.5 µL reactions, comprising of 5X Green GoTaq^®^ Flexi Reaction Buffer (Promega, Madison, WI, USA), 25 mM MgCl_2_ (Promega), 10 mM dNTP (Bioline, London, UK), 5mM of each primer (Metabion, Steinkirchen, Germany) and 1 unit of *Taq* polymerase (Go*Taq*; Promega). The use of Bovine Serum Albumin (BSA, 10 mg/mL) as an additive greatly improved the amplification yields of ITS and 28S fragments in all of the samples.

Conditions for polymerase chain reactions (PCR) for most ITS and 28S amplifications were: 3 min at 95 °C (denaturation), 35 cycles at 95 °C for 30 s (heating), 50 °C for 30 s (annealing) and 72 °C for 1 min (extension), followed by 72 °C for 5 min (final extension). However for several samples the following touchdown PCR programs proved to be most successful: 3 min at 95 °C (denaturation), 20 cycles at 95 °C for 30 s (heating), 55–45 °C (annealing; −0.5 °C per cycle) and 72 °C for 1 min (extension), followed by 20 cycles at 95 °C for 30 s (heating), 50 °C (annealing) and 72 °C for 1 min (extension), finally concluded by 72 °C for 5 min (final extension). Primer-dimers (short primer-primer fragments) and double bands in the PCR product were removed and purified with PEG (polyethylene glycol) cleanup or freeze squeeze extractions, with the latter one proving to be especially useful for multiple-band products (Tautz & Renz, 1983). Amplified and purified gene fragments were sequenced with BigDye^®^ terminator v3.1 (Applied Biosystems^®^) chemistry, following the manufacturer’s guidelines for Sanger sequencing technique at the Sequencing Service of the Department Biology, LMU—Genomics Service Unit in Martinsried, Munich on an ABI 3730 capillary sequencing machine. Sequences are deposited in the European Nucleotide Archive under accession numbers LS974447 –LS974515 and LS974852 –LS974856.

### Maximum likelihood and Bayesian inference analyses

After sequence correction and assembly with CodonCode Aligner (http://www.codoncode.com), the sequences were compared against NCBI Genbank (https://www.ncbi.nlm.nih.gov/genbank) using BLAST (Altschul et al., 1990) to check for possible contaminations. The data set was completed with other keratose sponge sequences as published in Genbank. Sequence alignment was conducted with MAFFT version 7.310 (Katoh & Standley, 2013). Alignments were post-processed by eye with Geneious version R8.1.9 http://www.geneious.com, (Kearse et al., 2012) and SeaView version 4.6.2 http://doua.prabi.fr/software/seaview, (Gouy, Guindon & Gascuel, 2010) due to the high variability of ITS regions. Ambiguous regions of the final alignments were excluded from the dataset using the Gblocks program (Castresana, 2000; Talavera & Castresana, 2007) as incorporated in SeaView, with options for ‘less strict flanking positions’ and ‘gap positions within the final blocks allowed’.

The relatively best-fitting models for phylogenetic reconstructions were calculated using jModelTest 2.1.9 (Darriba et al., 2012) for each dataset (ITS-2 and 28S). Phylogenetic reconstructions using Maximum Likelihood (ML) were conducted with RAxML 8.2.10 (Stamatakis, 2014), using the GTRGAMMAI model of nucleotide substitution for unpaired sites as suggested by the jModelTest results. Bayesian Inference (BI) reconstructions were performed with MrBayes 3.2.6 (Ronquist et al., 2012) under simultaneous runs of four Metropolis-coupled Markov chains (default temperature: 0.1) per Bayesian analysis using the most generalizing model (GTR+I+G) as possible, as overparametrization does not negatively influence Bayesian analyses (Huelsenbeck & Rannala, 2004). The analysis was set to 10,000,000 generations, with the chains stopping when the standard deviation of split frequencies reached values below 0.01. The sample frequency was set to every 500th generation.

## Results

### Morphology

**Figure 1:**
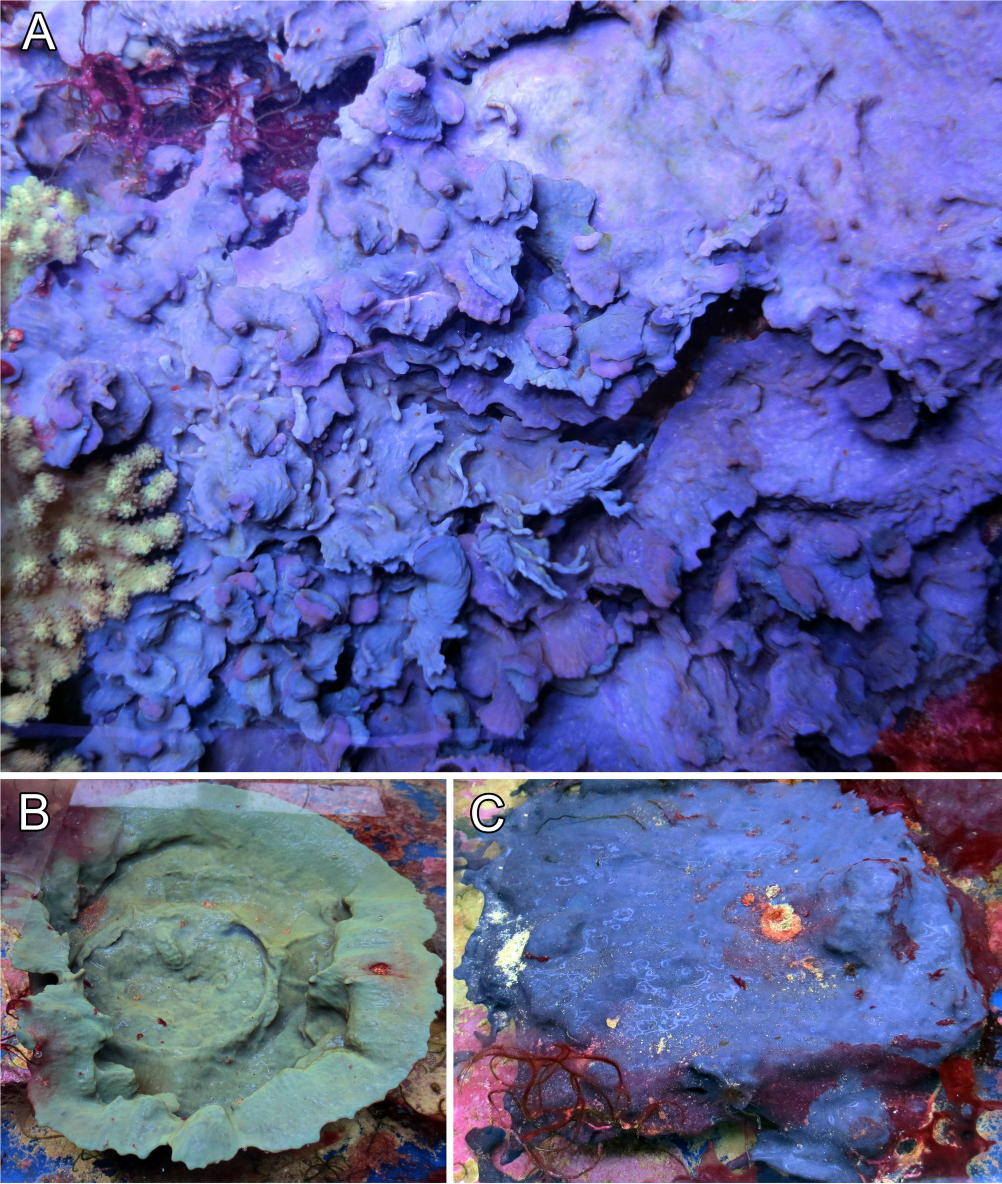
Growth form and color variations of the aquaria sponges, all with identical ITS genotype. (A) Foliose growth, two intermingled specimens of different purplish colors (GW8481); (B) cup shape, green color (GW30002); (C) encrusting growth, blue color (GW30003). All photos were taken from our laboratory aquaria.

The sponge’s growth form was variable and could range from a low spreading and encrusting form (see Fig. 1A, specimen GW8481), over a more foliose and lamellate growth (Fig. 1B, specimen GW30002) to a clear cup-shape (Fig. 1C, specimen GW30003). The surface was mostly smooth and glossy in appearance, and was usually free of any sand armour or encrustation. Oscules were very small (<1 mm) and were irregularly spread on the sponge’s surface, and again depending on the growth form, might not have been visible to the naked eye at all. The tissue was soft and flexible, but not very compressible in consistency. Similar to its growth forms, color was not consistent among individuals. While the two intertwined specimens in Fig. 1A were both of similar, but distinguishable purplish tint, the cup-shaped specimen (Fig. 1B) was of greenish color and the encrusting specimen (Fig. 1C) featured a bright blue hue.

**Figure 2:**
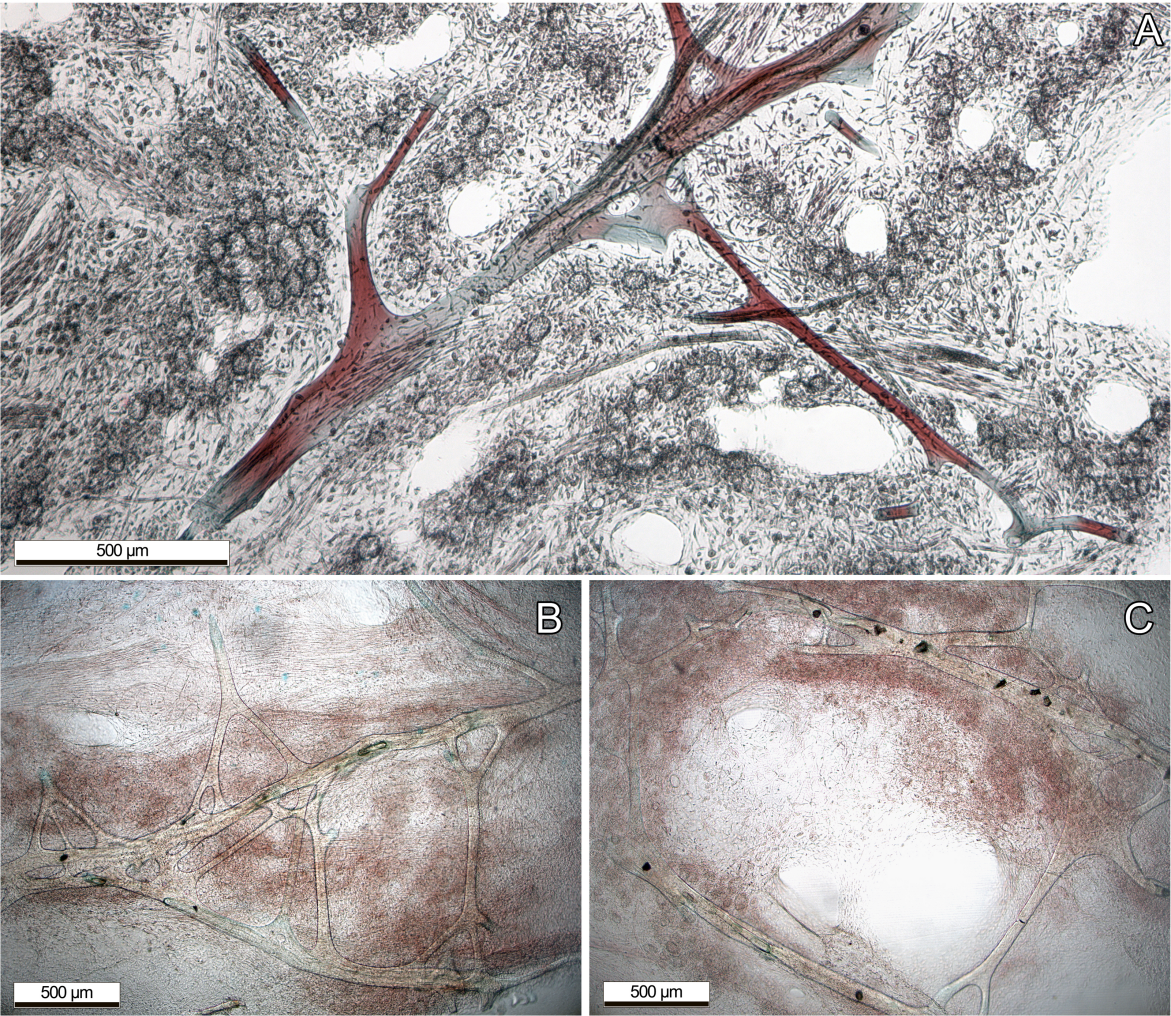
Dyed thin sections of the aquaria sponges. (A) Spongin fibres surrounded by choanocyte chamber clusters, channels, and collagenous tissue; (B) primary fibres with branching, not clearly distinguishable secondary fibres. (C) Primary fibres with coring of unknown foreign material, presumably sand.

Most information of internal structure and fibre composition were gained from surface parallel sections, implying that the majority of the skeleton has a similar orientation. These showed vast networks of irregularly branching primary, and secondary fibres. Fibers anastomosing from the secondaries were not clearly recognizable as tertiary fibers, as they were not clearly distinguishable (Fig. 2B). Only the primary fibers exhibited occasional, non-consistent coring with foreign debris. All fibers exhibited a fine lamination and hollow piths, although those were most notably in the large primary fibers. Choanocyte chambers of different sizes and shapes were randomly distributed across the sponge tissue, although sometimes forming loose clusters (Fig. 2A). The primary fibres of our examined specimens ranged from 90–140 µm diameter, the secondary fibers from 24–71 µm. Most of the choanocyte chambers were slightly oval shaped, ranging from 29.4 × 23.5 µm up to 76.5 × 58.8 µm. Both the fibres and chambers were embedded in a characteristic collagenous tissue (see Fig. 2A)

### Phylogenetic position of the aquaria sponge

**Figure 3:**
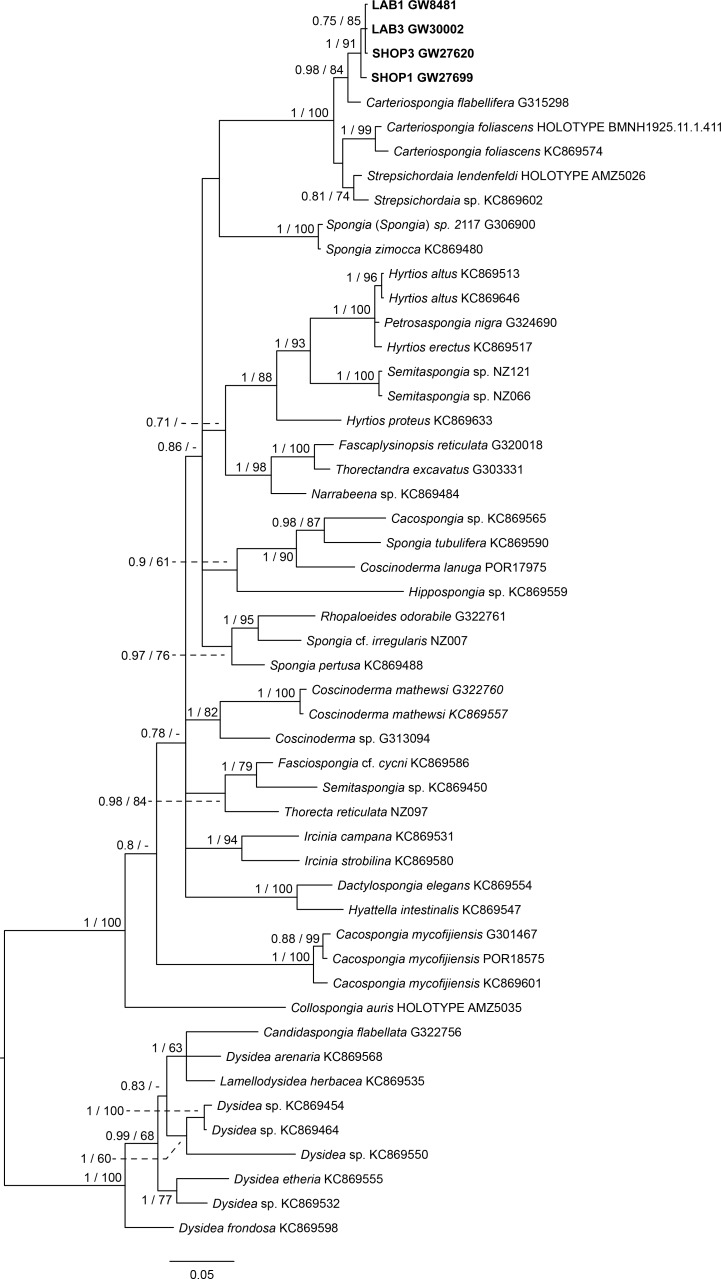
Bayesian inference phylogram of 28S of Phyllospongiinae and selected other Keratosa, rooted with dysideid outgroup taxa. Sequences of the targeted aquaria sponge are given in bold. Numbers following the taxon names represent museum collection numbers (i.e., QM Gxxxxxx, PORxxxx, SNSB- BSPG.GWxxxxx) or NCBI Genbank accession numbers. Numbers at the branches indicate Bayesian Inference posterior probabilities/corresponding Maximum likelihood bootstrap support.

**Figure 4:**
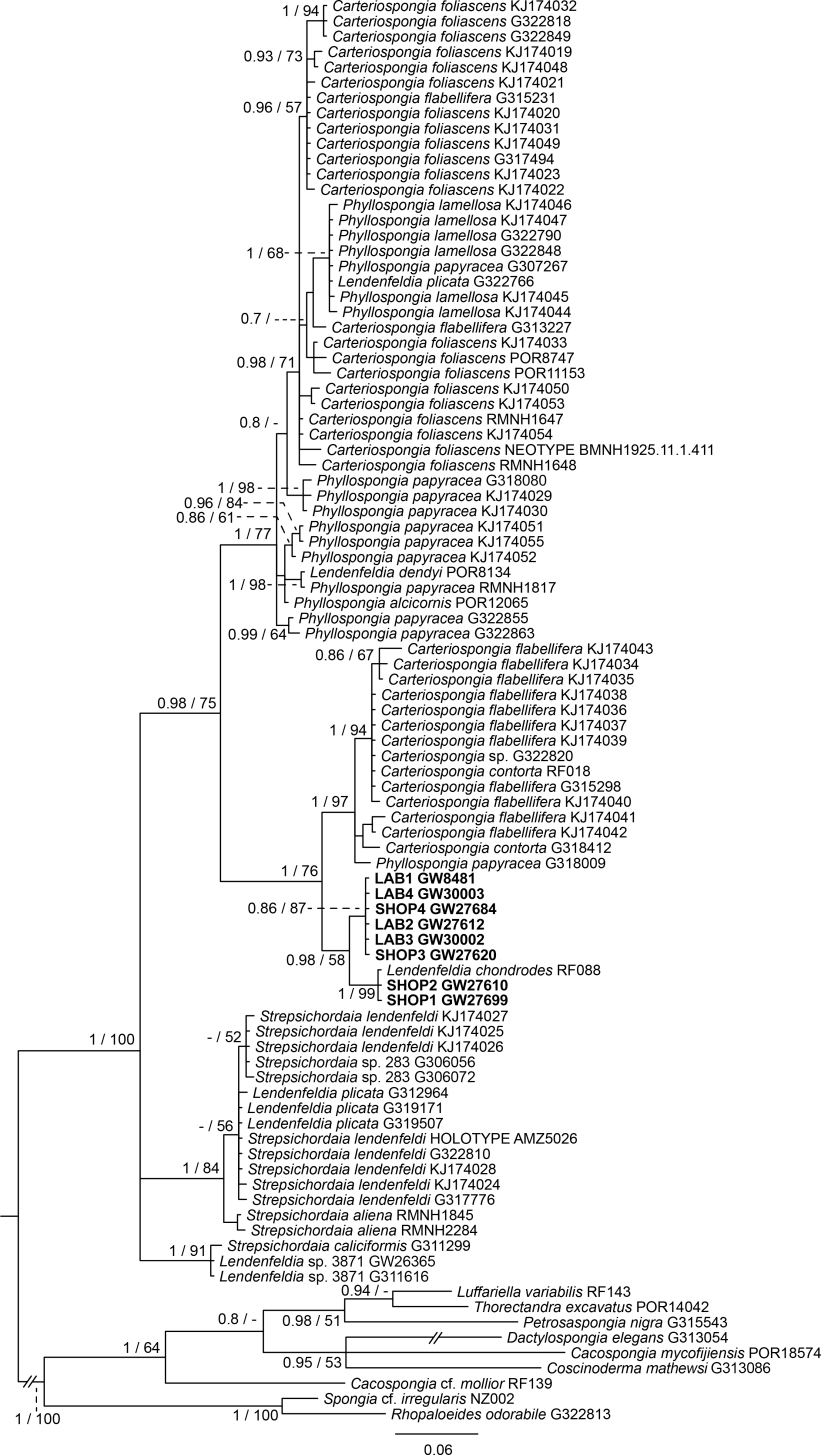
Bayesian inference phylogram of ITS2 of Phyllospongiinae, rooted with spongiid and thorectine outgroup taxa. Sequences of the targeted aquaria sponge are given in bold. Numbers following the taxon names represent museum collection numbers (i.e., QM Gxxxxxx, PORxxxx, SNSB- BSPG.GWxxxxx) or NCBI Genbank accession numbers. Numbers at the branches indicate Bayesian Inference posterior probabilities/corresponding Maximum likelihood bootstrap support.

The final 28S data set consisted of 51 taxa with 468 characters and contained 259 variable sites. The ITS2 data set for Spongiidae and Thorectidae consisted of 93 specimens with 248 characters and 192 variable sites. The 28S tree reveals a close relationship of the unidentified aquaria sponge to dictyoceratid sponges of the subfamily Phyllospongiinae (Family Thorectidae; Fig. 3). The four specimens formed a monophyletic sister to *Carteriospongia flabellifera*, and could further be divided into two distinct subclades. The sequence of the *Collospongia auris* holotype (AM Z5035), was distant to the aquaria sponge samples. The samples examined in our approach possessed two different genotypes (ITS-2, Fig. 4) with no difference between LAB and SHOP 3 and four specimens, but 8 bp between LAB and the mixed SHOP 1 and 2-*L. chondrodes* clade. The same two lineages could be identified with 28S, but with only 2 bp difference between LAB/SHOP3 and SHOP 1 specimens. Both markers reconstructed the aquaria sponges in a supported monophyletic group.

Based on the results as received from the 28S marker, the ITS data set was largely supplemented with the phyllospongiid data published in Abdul Wahab et al. (2014) (Fig. 4). The ITS-2 data was in congruence to 28S in respect to the position of the aquaria sponges distant to *Collospongia auris* in Phyllospongiinae. In contrary to the 28S a sequence of a *Lendenfeldia chondrodes* (De Laubenfels, 1954) (GW26715) could be included, which was a reference specimen (SDCC-RF088) for Cook & Bergquist (2002) for the Thorectidae chapter of the *Systema Porifera*, the currently most comprehensive revision of sponge genera. The *Lendenfeldia chondrodes* sequence falls into a clade with the aquaria samples. The aquaria samples (+*L. chondrodes* in ITS) clade is sister to *Carteriospongia flabellifera* and *C. contorta* in both 28S and ITS reconstructions.

### Phylogenetic position of remaining phyllospongiine taxa

The Phyllospongiinae *sensu* Cook & Bergquist (2002) do not form a monophyletic group. The *Candidaspongia flabellata* sequences in ITS and 28S reconstructions supported the placement of this species into the family Dysideidae, and distant to the phyllospongiine Thorectidae. The sequences of the remaining Phyllospongiinae, *Carteriospongia*, *Strepsicordaia*, *Phyllospongia* and *Lendenfeldia* form a well-supported monophyletic group within the Thorectidae. *Strepsichordaia,* also represented here by the holotype (AM Z5026) of the type species *S. lendenfeldi*, is the first branching species of the remaining Phyllospongiinae clade. *Carteriospongia* is a non-monophyletic genus, as *Carteriospongia foliascens,* type species of genus *Carteriospongia* Hyatt, 1877 and represented here by the neotype (BMNH 1925.11.1.411), is sister to *Phyllospongia papyracea* (type species of genus *Phyllospongia* Ehlers, 1870)*.* The remaining *Carteriospongia* species, *C. flabellifera* and *C. contorta* are sister to the aquaria samples (+ *L. chondrodes* in ITS) (see Fig. 4)*.*

## Discussion

### Identification as *Lendenfeldia chondrodes*, a phyllospongiine keratose sponge

The current study is the first time that a taxonomic identification of this well-known aquaria species has been attempted. Our molecular studies clearly reject any relationship to *Collospongia auris*, a species name frequently applied (e.g., Fosså & Nilsen, 1996; Osinga, Tramper & Wijffels, 1998; Brümmer & Nickel, 2003; Knop, 2016, and numerous aquaristic websites in the internet). Instead, molecular and morphological analyses clearly demonstrated a classification within the Phyllospongiinae Keller, 1889, which constitute shallow water sponges with a growth form allowing large surface areas exposed to the light (lamellate, vasiform, foliose) to accommodate photosynthetic symbionts (Wilkinson, 1988; Bergquist, Sorokin & Karuso, 1999). Macroscopic appearance in colour, surface structure and skeletal features in the specimens highly resemble *Lendenfeldia chondrodes* (see De Laubenfels, 1954; redescription in Bergquist, 1965). Our finding is supported by the molecular ITS-2 marker with specimens forming a clade with a *Lendenfeldia chondrodes* reference specimen from the *Systema Porifera*. Genetic differences in the highly variable ITS are in the range of genetic variation in other Phyllospongiinae lineages (see Abdul Wahab et al., 2014).

The origin of the misidentification of this aquaria species as *Collospongia auris* instead of *Lendenfeldia chondrodes* cannot be traced back with certainty. *Lendenfeldia* has repeatedly been confused with other keratose sponges (Bergquist, Sorokin & Karuso, 1999; Ridley et al., 2005 see discussions in Erpenbeck et al., 2012a; Thacker et al., 2013). This common confusion could presumably be traced back to high morphological plasticity, as its variations in growth and colour show (see Figs. 1 and 2). Different positions in the aquaria with exposure to different light and ambient water temperature conditions, as well as different substrates or nutrition appear to have a strong influence. Almost complete bleaching of the sponge is possible if kept under very low-light conditions for an extended period of time, thus a change in colour might also imply a change in symbiont or microorganism composition (S Vargas pers. comm., 2017). This effect, as well as its direct implications on the sponge itself, are currently under investigation. The physiological response of bleaching and recovery might however be similar to *C. foliascens*, as shown by Pineda et al. (2016).

Bergquist (1965) classified *L. chondrodes* into *Fasciospongia*, however Bergquist, Cambie & Kernan (1990) noted in their description of *C. auris* “Only one previously described species is similar in texture, gross morphology and fibre complement to *Collospongia auris* and that is *Fasciospongia chondrodes* from Palau”. In the same publication they re-classified *F. chondrodes* to *Lendenfeldia* based on fiber network and lamination different from *Fasciospongia* (Bergquist, Cambie & Kernan, 1990), consequently triggering a connection between *L. chondrodes* and *C. auris.*

### Phylogenetic implications of the molecular reconstructions

The molecular trees revealed current issues in the classification of the Phyllospongiinae, due to the polyphyly of the subfamily and paraphyly of its genera. Our data corroborate and extend earlier findings on the paraphyly of *Phyllospongia* and *Carteriospongia* (Erpenbeck et al., 2012b; Thacker et al., 2013; Redmond et al., 2013; Abdul Wahab et al., 2014). *Phyllospongia*, nominal genus of Phyllospongiinae (including *P. papyracea*, *P. alcicornis*, and *P. lamellosa*), and *Carteriospongia foliascens*, type species of *Carteriospongia,* form a clade with species of both genera mixed, and fell in a sister group relationship to *Carteriospongia flabellifera, C. contorta* and *Lendenfeldia chondrodes.* Consequently, revision and new classification of both genera is overdue (Abdul Wahab et al., 2014). Similarly, the taxonomic uncertainty also affects *Lendenfeldia* and the classification of the aquaria species *L. chondrodes*. In the present study, *L. chondrodes* is closely related to *Carteriospongia flabellifera*/*contorta*, but samples of *L. plicata* are distant and form a clade with *Strepsichordaia*. Consequently, the phylogenetic position of the *Lendenfeldia* type species, *L. frondosa* Lendenfeld, 1889, would be decisive to assign the correct *Lendenfeldia* in this polyphylum, but we did not manage to amplify a fragment from the holotype (BMNH 1877.5.21.1697). Therefore, *L. chondrodes* may potentially undergo a genus transfer in the future.

Molecular data of 18S rDNA (Redmond et al., 2013) and our 28S, ITS and CO1 data (see Supplementary file) clearly recover *Candidaspongia flabellata* (Bergquist, Sorokin & Karuso, 1999) within the Dysideidae. *Candidaspongia* is a monotypic genus and was placed in the Phyllospongiinae primarily by grounds of its scalarene compounds (Bergquist, Sorokin & Karuso, 1999). Although chemotaxonomy has been shown useful in many instances (see review in Erpenbeck & Van Soest, 2007), distinction of Phyllospongiinae and Dysideidae by sesterterpene compounds repeatedly resulted in obvious misidentifications (Jaspars et al., 1997; Bergquist, Sorokin & Karuso, 1999; see also Erpenbeck et al., 2012a) and requires re-assessment (Redmond et al., 2013). In addition the *Candidaspongia flabellata* spongin skeleton lacks the tertiary fibers which are typical for all other Phyllospongiinae. The placement of this genus into Dysideidae allows an amendment of Cook & Bergquist’s (2002) Phyllospongiinae definition as ‘folio-lamellate Thorectidae with tertiary fibres inthe skeleton’ without generic exception. Phyllospongiinae, therefore, only consists of the species currently assigned to the genera *Phyllospongia*, *Carteriospongia*, *Lendenfeldia* and *Strepsichordaia*.

## Conclusion

The identification of this common and easy culturable sponge as *Lendenfeldia chondrodes* instead of *Collospongia auris* opens up possibilities for further in-depth analyses on the production of bioactive compounds or other biological features in sponges. Phyllospongiinae possess a distinct suite of secondary metabolites and microbial symbionts, which now can be targeted precisely. Our molecular analyses underlines the importance of thorough taxonomic identification on the target organisms prior to every scientific study.

##  Supplemental Information

10.7717/peerj.5586/supp-1 Supplemental Information 1 Collection numbers (QM Gxxxxxx, PORxxxx, SNSB-BSPG.GWxxxxx, SDCC/xxxx, AM Zxxx ) or NCBI Genbank accession numbers of samples used for the reconstruction of the trees

10.7717/peerj.5586/supp-2 Supplemental Information 2 ITS data

10.7717/peerj.5586/supp-3 Supplemental Information 3 28S data

## Additional Information and Declarations

### Data Availability

The following information was supplied regarding data availability: Sequences are deposited in the European Nucleotide Archive under accession numbers LS974447-LS974515 and LS974852-LS974856.
